# Medical Applications of Molecular Biotechnologies in the Context of Hashimoto’s Thyroiditis

**DOI:** 10.3390/diagnostics13122114

**Published:** 2023-06-19

**Authors:** Maria Trovato, Andrea Valenti

**Affiliations:** Department of Clinical and Experimental Medicine, University Hospital, 98125 Messina, Italy

**Keywords:** Hashimoto’s thyroiditis, molecular biotechnologies, clinical trials studies, hygiene hypothesis, parvoviruses

## Abstract

Hashimoto’s thyroiditis (HT) is a gender autoimmune disease that is manifested by chronic inflammation of the thyroid. Clinical trial studies (CTSs) use molecular biotechnologies (MB) to approach HT appearance. The aims of this study were to analyze the applications of MB in CTSs carried out in HT populations (HT-CTSs). Further, to evaluate the role of MB in the context of the hygiene hypothesis (HH). From 75 HT-CTSs found at clinicaltrials.gov web place, forty-five were considered for this investigation. Finally, six HT-CTSs were reported as molecular HT-CTSs (mHT-CTSs) because these were planning to utilize MB. Two of mHT-CTSs were programmed on the French population to isolate DNA viral sequences. Blood, urine, and thyroid tissue biospecimens were analyzed to pick out the parvo and polyoma viruses. Two mHT-CTSs carried out in China aimed to identify oral and fecal microbiotas by measuring PCR sequencing of the 16S rRNA gene. Two mHT-CTSs were programmed in the USA and Greece, respectively, for interception of DNA polymorphisms to associate with genetic susceptibility to HT. In conclusion, MB are mainly employed in HT-CTSs for infective pathogenesis and genetic fingerprinting of HT. Furthermore, MB do not provide evidence of HH; however, they are useful for providing direct evidence of the presence of viruses.

## 1. Introduction

Hashimoto’s thyroiditis (HT) is a chronic destructive inflammatory process that develops by autoimmune mechanisms [1]. HT falls within autoimmune thyroid diseases (AIDT) precisely because of the inflammatory response to immune alterations [2,3]. 

Basically, morphological features of HT include four signatures such as lymphoid infiltrates, fibrosis, oxyphilic changes of follicular cells and varying degree of destruction of glandular tissue [4]. 

Immunological hallmarks of HT enclose serum antibodies raised against various thyroid antigens encompassing from thyroid peroxidase and antithyroglobulin to thyroid-stimulating hormone receptors [5,6]. Morphological and immunological HT traits do not emerge concurrently. In fact, there is a small proportion of patients that show cytological features of HT, whereas thyroid antibodies are lowly detectable in their serum [7]. 

Reductions of serum thyroid hormone levels are noted in HT patients: this is in case no glandular cells secrete enough thyroid hormones able to meet the needs of the body. However, hypothyroidism (hy-T) is diagnosed based on serum levels of several biochemical markers such as thyroid stimulating hormone (TSH) and other thyroid hormones used to confirm the diagnosis [8,9]. HT hormonal indicators report different degrees of hy-T, independently according to the existence of morphological damage [4]. Therefore, HT may clinically present either with prominent or mild hy-T symptoms [1]. These appear related to the atypic activity of muscle and nerve fibers, the alteration of glucose-lipid metabolism, and cognitive and psychological disorders [10,11,12]. 

Currently, a biochemical “grading” system is used to identify latent hy-T forms (see Table 1 in Ref. [4]) [4]. Above all, this system is designed for HT treatment by levothyroxine (L-T4). This system is built by the confrontation between serum levels of TSH and free thyroxine (T4). At the time that L-T4 replacement was indicated as the first choice for the treatment of hy-T, the method of performing this hormonal comparison became essential. In fact, since 2014, the guidelines of the American Thyroid Association have recommended the L-T4 treatment strategy for hy-T forms [13,14,15,16]. Further, this is in accordance with nationwide data from the National Health Service in the United Kingdom and the European thyroid association [17]. However, basic science and clinical evidence are inducing the development of investigations on LT4/LT3 combination therapy [18,19]. New data has come up regarding the limitations of serum TSH biochemical markers because they partially reflect the patient’s total thyroid status [20,21,22,23,24]. Lastly, 10–15% of hy-T patients voiced their discontent because of L-T4 treatment outcomes [22,23]. In fact, this recent evidence urges the involvement, in the care of hy-T, above all, of hy-T patients themselves [22,23,24,25].

### 1.1. HT Biomarkers and Epidemiological Data

Overall, HT diffusion can be reported by considering two distinct types of serum biomarkers. Firstly, HT epidemiological information can be compiled based on autoimmune biomarkers of thyroid inflammation. Secondly, to archive HT epidemiological data, HT diffusion can be related to biochemical markers of hy-T and then to the onset of hy-T symptoms.

By focusing on serum immunological biomarkers, HT is considered a gender-functional disorder [26]. This is because of the mechanisms underlying the appearance of autoantibodies [27,28]. In fact, the disruption of immune tolerance is genetically driven [29,30]. Particularly, HT autoimmune anomalies are genetically based on gender and pre-existing individual susceptibility [27,29,30]. In turn, the environment plays a critical role in altering genetic backgrounds by influencing disease development [26,30]. Hence, HT is reported in women 10–15 times more often than in men, with an incidence peak at around 30–50 years of age [31]. Conversely, in men, the HT incidence increases with aging and the incidence peak is reached 10–15 years later [31]. 

When HT diffusion is related to hy-T incidence, substantial differences emerge between hy-T that spreads in endemic areas of iodine deficiency and that which is accompanied by HT. Zimmermann and colleagues previously observed that hy-T typically emerges in HT patients independently according to iodine nutrition status [32,33]. This is because hy-T develops even in HT patients living in areas with sufficient iodine intake. Moreover, when populations are resident in iodine-deficient localities, it is quite common to find endemic hy-T [34]. 

Gender differences come up even when HT is related to the onset of hy-T signs and symptoms. In fact, distinctive clinical courses and different outcomes are observed in women compared to men. Further, differences in gender are a key determinant even of therapeutic responses to L-T4 [26,35]. Usually, hy-T symptoms include fatigue, cold intolerance, and constipation. However, there is a large variation in the clinical presentation of symptoms [15].

In women, hy-T develops more frequently at a later age than HT, especially after 60 years of age [26]. In addition, hy-T symptoms have no determinant role in the identification of endocrine disorders. This is because hy-T symptoms may occur in healthy female subjects, too. Lastly, L-T4 therapy may be associated with residual symptoms despite normal thyroid tests [17,24]. 

In men, hy-T symptoms that accompany overt HT are more recurrent, last longer, and are usually less treatable [26]. Therefore, the presence or absence of symptoms may be contributing factors in the identification of hy-T. Lastly, L-T4 therapy is less frequently accompanied by other side effects in men.

### 1.2. Prevalence of HT Diagnoses

The methods used to diagnose HT have a long history related to the description of morphological alterations of the thyroid gland, the recognition of autoimmune pathogenesis, and the identification of thyroid hormones [4]. For a proper diagnosis of HT, several methods are involved; further, different biomarkers are assessed independently or in combination with each other [4]. Mainly, serum, ultrasound, and pathological examinations are considered HT diagnostic methods [4,31]. Current research has reported the global prevalence of diagnoses of HT according to different diagnostic methods [31]. Moreover, data about the prevalence of methods useful to confirm HT diagnoses have been provided [31]. Therefore, HT is prevalently diagnosed by ultrasonography (13.2%) and pathological examination (12.5%) [31]. When the serum autoantibody profile is considered, the prevalence rate of HT diagnosis stands at 7.8% (see Figure 9 in Ref. [31]) [31]. The combination of two methods, including serum antibody titers and color Doppler ultrasound, is used for HT diagnosed with a prevalence of 10.4% [31]. This prevalence is considerably lower (4.7%) if three methods, such as autoantibody titers, color Doppler ultrasonography, and fine needle aspiration, are combined. To confirm HT diagnosis, thyroid tissue alone is prevalently used (14.1%) [31].

### 1.3. Molecular Biotechnologies (MB) and HT

MB are pivotal for new biomedical methodologies because of their capability to reveal molecular pathogenetic pathways as well as the genetic susceptibility of populations to develop AIDT [6]. Above all, now the use of genetic analysis is turning out to be a key tool for clinical genomic investigations owing to its high accuracy, reproducibility, and reliability of results. 

The effective clinical application of MB can be assessed in accordance with the advice of qualified clinical trial studies (CTSs) [36]. These investigations are the basis of genomic screenings designed to detect viral genetic material involved in the pathogenesis of diseases. Not only this, but CTSs can especially test how well genomic screenings work to identify susceptibility to develop diseases in subgroups of populations belonging to a specific continent.

Molecular alterations occurring in the context of HT play crucial roles in promoting the cellular proliferation of both lymphocytes and glandular tissue. Indeed, mucosa-associated lymphoid tissue (MALT) lymphomas can originate at the site of HT [37,38]. On the other hand, for a considerable time, HT was reported concurrent with cancerous follicular lesions such as nodular goiter, adenoma, and carcinoma [38,39,40,41]. MB are currently employed for the classification of MALT lymphoma [42,43]. Further, these analyses are applied in dubious diagnoses of thyroid glandular cancerous lesions [44]. Mainly, these are part of innovative thyroid medicine that aims through biomarkers to early molecular diagnosis, personalized treatment, the prediction of cancerous risk, and prognostic information [45]. 

There were two main objectives of the study: firstly, to perform a systematic analysis of CTSs conducted on HT populations living at different geophysical latitudes (HT-CTSs). This was done to establish the frequency with which these CTSs were concluded on different continents and when they were planned. Secondly, to identify samples in which MB were applied. 

Therefore, a wide-ranging search was conducted on CTSs provided at https://beta.clinicaltrials.gov/ (accessed on 16 April 2023) through the files covered by “autoimmune thyroiditis Hashimoto” keywords [46]. Following this, some of these findings were selected as they referred to HT-CTSs that planned to apply molecular technologies (mHT-CTSs).

In the context of the hygiene hypothesis (HH), divergences among geographic diffusion of HT and molecular fingerprint of HT patients were also considered.

The current applications of MB for pathological practices were discussed separately. Mainly, these concern molecular aspects for the diagnosis of malignant thyroid lesions associated with HT. 

## 2. Material and Methods

### 2.1. Data Sources 

A systematic review of CTSs for HT was performed by surveying all the results of the search for the term “autoimmune thyroiditis Hashimoto,” namely under “Condition or disease” at https://beta.clinicaltrials.gov/ (accessed on 16 April 2023) [46]. 

### 2.2. Study Selection 

Seventy-five CTSs were found using these keywords that also included 3 synonyms of conditions or diseases such as “autoimmune thyroiditis,” “thyroiditis Hashimoto,” and “Hashimoto” [46]. Mainly, 29 related terms were found, of which 10 pertained to “autoimmune thyroiditis” synonyms (Hashimoto, thyroiditis autoimmune, Hashimoto disease, HASHIMOTO THYROIDITIS, Hashimoto’s thyroiditis, Hashimoto’s disease, chronic thyroiditis, Hashimotos disease, chronic lymphocytic thyroiditis, and lymphocytic thyroiditis), ten to “thyroiditis Hashimoto” (Hashimoto, Hashimoto disease, autoimmune thyroiditis, HASHIMOTO THYROIDITIS, Hashimoto’s thyroiditis, Hashimoto’s disease, chronic lymphocytic thyroiditis, chronic thyroiditis, lymphocytic thyroiditis, Hashimotos disease), and 9 to “Hashimoto” (Hashimoto disease, autoimmune thyroiditis, HASHIMOTO THYROIDITIS, Hashimoto’s thyroiditis, Hashimoto’s disease, chronic lymphocytic thyroiditis, chronic thyroiditis, lymphocytic thyroiditis, and Hashimotos disease) [46].

### 2.3. Inclusion Criteria 

There were two inclusion criteria adopted for this investigation. The enrollment of HT patients was designed as the first criterion for selecting CTSs, whereas the molecular analyses were used as the second.

In the first instance, the above CTSs were scrutinized to enucleate the full set of trials carried out on HT populations. Secondly, to assess the effective application of MB, the “Study Plan” sections were analyzed for all CTSs [46]. Here, there were details on how a single CTS was planned and what the study was measuring. Particularly, the “Outcome Measure” sub-section provided insight into the use of molecular analysis to realize the aim of the CTS. 

### 2.4. Exclusion Criteria 

Upon reviewing and studying the 75 CTSs, 30 of them were considered irrelevant. Mainly, 5 CTSs were eliminated from this study because they did not recruit participants with HT: i.e., HT was a “Medical Subject Headings” term or a collateral effect to a therapy (NCT05077865, NCT04239521, NCT04349761, NCT05680376, NCT03957616).

Twenty-four CTSs remained outside because these concerned other autoimmune, inflammatory, or lymphocytic diseases (NCT03872284, NCT04823728, NCT05225883, NCT03993262, NCT05177939, NCT04339205, NCT04175522, NCT03530462, NCT03835728, NCT03542279, NCT03004209, NCT05198661, NCT04106596, NCT04708626, NCT01456416, NCT04875975, NCT05280600, NCT05682482, NCT05605223, NCT05503264, NCT03941184, NCT05422664, NCT05711563, NCT05772611).

One CTS was omitted because the “Hashimoto” keyword indicated the location of the study (NCT04339127).

### 2.5. Data Extraction 

According to the first inclusion criteria, data extraction was performed. Basically, 45 CTSs were extracted for evaluation and included in this study because they had effectively investigated HT populations (Table 1). These HT-CTSs were entered into systematic analysis by evaluating 7 variables such as the continent and geo-location, the start date, the primary completion date, the completion date, the last verified, and the conclusion of the study (Table 1). 

**Table 1:** Clinical trial studies conducted on the HT population.

Continent	Geolocation	ClinicalTrials.gov Identifier	Official Title	Start Date *	Primary Completion Date *	Study Completion Date *	Last Verified	Conclusion
Africa (*n* = 2) 0.04%	Egypt	NCT03289403	The role of immunomodulatory treatment in the success of ICSI in patients with autoimmune thyroiditis	2018	2019	2019	2020	Completed
	Israel	NCT01270425	Sonographic and laboratory evaluation of the thyroid gland in patients with systemic sclerosis	2011	2011	2012 (anticipated)	2013	Completed
American (n = 7) 15.5%	Brazil	NCT01129492	Low-level laser therapy in chronic autoimmune thyroiditis	2006	2009	2009	2010	Completed
	Brazil	NCT02240563	Low-level laser therapy for autoimmune thyroiditis	2014	2016	2016	2017	Completed
	Chile	NCT04778865	Effect of treatment for vitamin D deficiency on thyroid function and autoimmunity in Hashimoto’s thyroiditis	2020	2021 (estimated)	2021 (estimated)	2021	Recruiting
	USA	NCT00958113	Autoimmune thyroid disease genetic study	2009	2013	2015	2015	Completed
	USA	NCT01428167	Hashimoto’s thyroiditis and thyroid cancer (thyroid cancer)	2011	2012	2012	2012	Completed
	USA	NCT01551498	Evaluating the dietary supplement Anatabloc in thyroid health ASAP (Anatabloc supplementation autoimmune prevention; ASAP)	2012	2013	2013	2015	Completed
	USA	NCT04542278	Preoperative steroids in autoimmune thyroid disease	2020	2022	2022	2022	Completed
Asian (n= 6) 13.3%	China	NCT03447093	The oral microbiota is associated with autoimmune thyroiditis	2017	2019 (estimated)	2021 (estimated)	2018	Unknown
	China	NCT04075851	The prevalence of serum thyroid hormone autoantibodies in autoimmune thyroid diseases	2019	2022 (estimated)	2022 (estimated)	2021	Recruiting
	China	NCT04942769	Study on the effect of selenium supplementation on the structure and function of autoimmune thyroiditis	2019	2021 (estimated)	2021 (estimated)	2021	Recruiting
	China	NCT03390582	Gut microbiota is associated with autoimmune thyroid disease	2017	2018 (estimated)	2021 (estimated)	2018	Unknown
	Taiwan	NCT02126683	The effect of Plaquenil on serum inflammatory markers and goiter in euthyroid young women with Hashimoto’s thyroiditis	2014	2016 (estimated)	2016 (estimated)	2014	Unknown
	Taiwan	NCT01760421	The effect of hydroxychloroquine treatment in Hashimoto’s thyroiditis	2011	2012	2013	2014	Completed
Europe (n = 22) 48.8%	Denmark	NCT02013479	Selenium supplementation in autoimmune thyroiditis (CATALYST)	2014	2022	2022 (estimated)	2022	Active, not recruiting
	France	NCT03114267	Involvement of viral infections in the pathogenesis of chronic lymphocytic thyroiditis (Etude thyrovir)	2012	2015	2015	2017	Completed
	France	NCT03103776	Involvement of polyoma viruses in pathogenesis of autoimmune thyroiditis and goitrigenesis (IPoTAIG)	2016	2018 (estimated)	2018 (estimated)	2018	Unknown
	France	NCT04789993	Additional autoimmune diseases with type 1 diabetes in pediatric patients at diabetes diagnosis and during follow-up (AADT1D)	2021	2021 (estimated)	2021 (estimated)	2021	Enrolling by invitation
	France	NCT05544448	In vitro effect study of interleukin-2 muteins on regulatory T cells of patients with different autoimmune, alloimmune, or inflammatory diseases (MuTreg)	2022	2023 (anticipated)	2023 (anticipated)	2022	Not yet recruiting
	Germany	NCT00552487	Isolated ACTH deficiency in patients with Hashimoto’s thyroiditis	2005	NA	2006	2007	Completed
	Greece	NCT02491567	DNA methylation and autoimmune thyroid diseases (THYRODNA)	2014	2016	2018	2019	Completed
	Greece	NCT02644707	Selenium supplementation in youths with autoimmune thyroiditis (THYROSEL)	2014	2016	2018	2020	Completed
	Greece	NCT04693936	Metabolic biomarkers in Hashimoto’s thyroiditis and psoriasis	2021	2023 (estimated)	2024 (estimated)	2022	Recruiting
	Greece	NCT02725879	FGF-21 levels and RMR in children and adolescents with Hashimoto’s thyroiditis (THYROMETABOL) (THYROMETABOL)	2016	2020 (estimated)	2020 (estimated)	2020	Unknown
	Italy	NCT03498417	Anti-insulin-like growth factor- 1 receptor (IGF-1R) Antibodies in Graves’ Disease and Graves’ orbitopathy (IGF1RAbsGO)	2018	2018	2018	2018	Completed
	Italy	NCT01465867	Selenium supplementation in pregnancy (Serena)	2012	2017	2018	2018	Completed
	Norway	NCT02319538	Hashimoto—a surgical disease, total thyroidectomy makes antibodies disappear and ameliorates symptoms	2012	2017	2017	2018	Completed
	Poland	NCT04752202	The influence of reducing diets on changes in thyroid parameters in obese women with Hashimoto’s disease	2019	2019	2019	2021	Completed
	Poland	NCT04682340	Analysis of BPA concentration in serum in women of reproductive age with autoimmune thyroid disease	2020	2021	2022	2022	Completed
	Romania	NCT04600349	Identity oriented psychotrauma therapy on Hashimoto in adults	2020	2020	2021	2021	Completed
	Romania	NCT04472988	Eye movement desensitization and reprocessing on autoimmune thyroiditis in adults	2020	2020	2021	2021	Completed
	Switzerland	NCT05017142	Swiss pediatric inflammatory brain disease registry (Swiss Ped-IBrainD)	2020	2071 (estimated)	2071 (estimated)	2021	Recruiting
	Turkey	NCT01102205	Evaluation of oxidative stress and the effect of levothyroxine treatment on oxidative stress in Hashimoto’s disease	2010	2010	2010	2013	Completed
	Turkey	NCT04754607	Effects of low-level laser therapy on oxidative stress levels, fatigue and quality of life in patients with Hashimoto’s thyroiditis	2021	2022	2022	2022	Completed
	Turkey	NCT00271427	Selenium treatment in autoimmune thyroiditis (AIT)	2004	NA	2005	2006	Completed
	Turkey	NCT01644318	CXCL9 and CXCL11 levels in patients with autoimmune thyroiditis and habitual abortions	NA	NA	NA	2012	Unknown
Unknown (n = 8) 17.7%	Not provided	NCT01884649	Fetuin-A as a new marker of inflammation in Hashimoto’s thyroiditis	2012	2012	2012	2013	Completed
	Not provided	NCT02318160	Oxidative status in children with autoimmune thyroiditis	2014	2014	2014	2014	Completed
	Not provided	NCT04613323	Management of thyroid function in Hashimoto’s thyroiditis during pregnancy	2022 (estimated)	2022 (estimated)	2022 (estimated)	2021	Not yet recruiting
	Not provided	NCT02190214	Thyroid disorders in Malaysia: a nationwide multicentre study (MyEndo-Thyroid)	2014	2016	2016	2016	Completed
	Not provided	NCT03048708	Thyroid in bariatric surgery (ThyrBar)	2011	2013	2016	2018	Completed
	Not provided	NCT02302768	Effect of Semet (80 and 160 mcg) versus placebo in euthyroid patients with AIT	2012	2014	2015 (estimated)	2014	Unknown
	Not provided	NCT05435547	Preoperative corticosteroids in autoimmune thyroid disease	2022	2025 (anticipated)	2025 (anticipated)	2022	Not yet recruiting
	Not provided	NCT05276063	A Phase 2b, study of Linsitinib in subjects with active, moderate to severe thyroid eye disease (TED; LIDS)	2022	2023 (estimated)	2025 (estimated)	2023	Recruiting

* NA: not available; data taken from reference [46].

### 2.6. Data Synthesis

According to both inclusion criteria, data were synthesized. Finally, 6 mHT-CTSs were recorded because they had scheduled to use MB to realize their aims (Table 2). To evaluate each mHT-CTS, 6 additional variables were added to the previous 7. These corresponded to target sequences, analysis and methods, biospecimen genetic retention and description, type and model of the study, time perspective, and the enrollment of subjects, respectively. The responsible party and results overview are shown in Table 2. 

## 3. Results

### 3.1. CTSs Conducted on HT Population

Forty-five CTSs enrolled HT patients. Thirty-seven of them provided information about geolocations by specifying where studies have been conducted (Table 1). In fact, there were no items in 17.7% of HT-CTSs (Table 1). 

HT-CTSs were geographically assigned to four continents with different distributions. Then, 0.04% of HT-CTSs were conducted in Africa, 15.5% in America, 13.3% in Asia and 48.8% in Europe (Table 1). 

In Africa, HT-CTSs were planned between 2011 and 2018. In America, HT populations have been listed in CTSs since 2006, whereas in Asia, this was done from 2011 onwards. In Europe, the first CTS on the HT population was arranged in 2004 (Table 1). 

Both HT-CTSs planned in Africa were completed (Table 1). Out of a total of 7 HT-CTSs mapped on the American continent, around 85.7 percent were completed. Conclusions were found only in one of six Asian HT-CTSs (16.6%) and in 14 of 22 European HT-CTSs (63.6%; Table 1). 

These data indicate that HT-CTSs especially provide a large amount of information about populations living at European latitudes. This is due to the hugest number of planned and concluded HT-CTSs in Europe with respect to other continents. 

### 3.2. Clinical Application of MB in HT-CTSs 

In the list of mHT-CTSs, six trials were included. Two of them (33.3%) were finalized to display viral sequences. For the four remaining HT-CTSs, two were designed to identify bacteria and two to set genetic polymorphisms to associate with susceptibility for HT (Table 2). 

Two DNA viruses were investigated from mHT-CTSs in the French population: these corresponded to the parvo and polyoma viruses (Table 2). Both viruses were identified by the polymerase chain reaction (PCR) method. Especially, genetic strands of polyoma virus were detected in different biospecimens such as blood, urine, and thyroid tissues. Based on the study model, the spread of parvovirus was screened through an observational study (NCT03114267). Conversely, the polyoma virus was approached by an interventional CTS (NCT03103776). A cohort model with retrospective analysis was followed for the observational study. Contrariwise, a parallel assignment model was assigned to interventional HT-CTSs (Table 2). Consequently, both mHT-CTSs were planned to have knowledge about the viral pathogenesis of HT by PCR analysis. However, the NCT03114267 CTS investigated viral causes and effects using a longitudinal analysis that retrospectively evaluated the outcome in an HT population. In contrast, the NCT03103776 CTS investigated viral causes and effects on several populations affected by autoimmune diseases, among which was an HT population. 

Two were the mHT-CTSs investigating bacteria, which aimed at identifying microbiotas. Oral and fecal microbiota were examined in the Chinese population by measuring PCR sequencing of the 16S rRNA gene (Table 1 and Table 2). Human feces were used to pick up microbiota genetic materials for the investigation, namely NCT03390582 (Table 2). Both mHT-CTSs were observational studies; however, oral microbiota was evaluated by a case-control study, whereas fecal microbiota was evaluated by a cohort study (Table 2).

Among mHT-CTSs programmed for interception of HT susceptibility, the NCT00958113 investigation was performed in Colorado (USA), whereas the NCT02491567 CTS focused on the Greek population (Table 1 and Table 2). DNA was examined on biospecimens such as saliva and blood leucocytes. Both mHT-CTSs pertained to observational, case-control studies with cross-sectional examinations.

## 4. Discussion

HT may appear through different clinical and histological aspects, and thus, morphological and serum diagnoses of HT are not equivalent [4]. In addition, HT may be associated with benign and malignant follicular lesions as well as lymphomatous proliferations [38,41,42]. The exact etiology of HT still remains incompletely elucidated. Mainly, it has been related to interactions of different elements, such as genetic alterations, environmental and epigenetic factors [30,47,48]. MB are promising surveying methods to apply to the HT population. 

Totally, 75 CTSs were examined in this study to assess the effective clinical use of MB for planning trials. Through the examination of mHT-CTSs, it has emerged that MB have been employed for two unique purposes. Firstly, to reveal infective etiopathogenesis of HT and, secondly, to determine molecular fingerprinting of HT in populations. Mostly, in this investigation, four trials were isolated in which clinical applications of MB served to display viral or bacterial genomes. This demonstrates how these methods are properly functioning to explore the complexity of infective HT pathogenesis. 

Viral and bacterial infections are currently involved in HT pathogenesis via multiple and often intertwined pathways. 

Based on the old Th1/Th2 paradigm, the so-called hygiene hypothesis (HH) has been adapted to the infective etiology of AIDT at the end of the last century [49,50,51,52]. Briefly, this hypothesis postulates that early infections in childhood protect against the establishment of autoimmunity [49,52,53,54,55]. Further, reduced exposure to microbial environments in childhood is considered an element conducive to the increase of autoimmune diseases in adults [56]. This is because an immune system educated by pathogen exposition may better suppress autoimmunity. However, the extension of HH to support HT pathogenesis has not reported a complete agreement [52]. 

Closely related to HH are the socio-demographic profiles of the HT population, data from migration surveys and biographic info of HT patients. 

**Figure 1:**
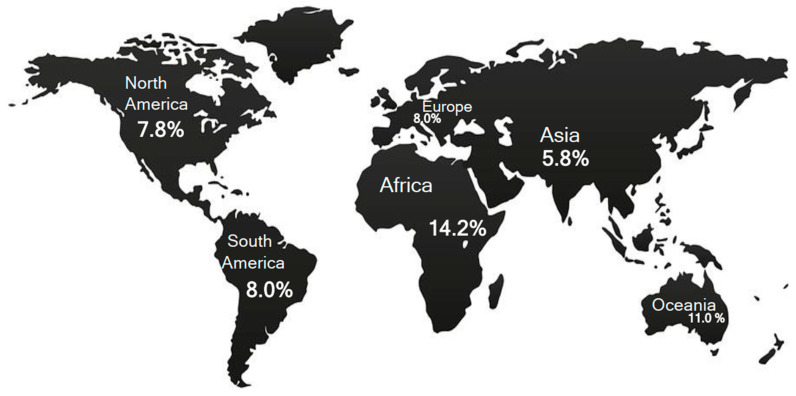
Global HT prevalence. Data has been extracted from reference [31].

By different concentrations, HT subjects are geographically distributed on the continental territories. A geographical map created on the bases of demographic observations reveals higher concentrations of HT subjects in Africa and Oceania (Figure 1) [31]. On the basis of socio-demographic observations, two divergent findings have been recorded. In low- and middle-income countries, the highest prevalence of HT patients is found among low-middle-income subjects (11.4%; see Figure 8 in Ref. [31]) [31]. However, HT patients are prevalently concentrated in high-income countries [31]. Therefore, the HH pathogenetic concepts can be applied to the last phenomena, whereas the first evidence seems limited only to the infectious etiology of HT. 

For over 50 years, surveys on the transmigration of populations are persistently reporting that subjects migrating from a country with a low incidence of autoimmune disorders develop immune-related diseases with the same frequency as the original inhabitants of the host country [53,57,58,59,60,61,62]. These data suggest an environmental effect at the beginning of autoimmune diseases. 

By reporting the biographic info of HT patients, several investigations have focused on the surprising association occurring between the birth month of individuals and HT. Mostly, HT patients were born in winter and autumn [63]. This data suggests that cold weather protects against TPO-Ab development [64]. Nevertheless, this evidence is consistent with the infective etiology of HT due to the abundant spread of infectious agents in winter. Further, these findings support HH because children born in winter have early exposure to infectious agents, facilitating the development of autoimmune diseases. However, moving from these premises, it is even possible to affirm that the incidence of HT for the individual subject may be predicted based on their date of birth. Summing up these phenomena, HH seems jarring with genetic features observed in autoimmune disorders, especially in HT. 

Molecular analyses have mapped on the short arm of chromosome 6 (6p) a super-region of 7.6 Mb, including the extended major histocompatibility complex (eMHC) [65,66]. This region lengthens telomerically from RPL12P1 to HIST1H2AA, and it is composed of six clusters and six super-clusters [66]. At 6p21.3 of eMHC, human leukocyte antigen (HLA) genes are localized, which are highly polymorphic [66]. HLA expressions are strongly related to infection, immunity, and inflammation [67].

In HT, genetic polymorphisms of HLA change depending on ethnicity [68]. This is because of different expressions of haplotypes in Caucasians (DR3, DR5, DQ7, DQB1*03, DQw7 or DRB1*04-DQB1*0301) with respect to Japanese (DRB4*0101, HLA-A2, DRw53) and Chinese (DRw9) HT patients [68]. Together, this data suggests that non-genetic factors trigger the onset of autoimmune disorders through an unidentified genetic background that is common to the entire HT population. Therefore, among phases composing HT pathogenesis, individual genetic susceptibility enters at a later stage than environmental factors.

Genetic disparities in HLA profiles are established through the use of molecular techniques. These methods have the advantage of systematically arranging HLA haplotypes using symbols. The complexity of the nomenclature of HLA haplotypes has been organized using multiple molecular techniques [69]. The first molecular approach to displaying HLA alleles concerned the application of Sanger sequencing-based typing (PCR-SBT) methods [69]. High-throughput sequencing (HTS) methods, including next-generation “short-read” (NGS) and third-generation “long-read” sequencing methods, are the natural evolution of PCR-SBT. Lastly, Oxford Nanopore Technology MinION is progressively reorganizing the number of HLA alleles [70]. Genotyping investigations on Graves’s disease (GD) have identified novel HLA alleles through high-resolution NGS [71,72]. Further, methods based on machine learning are useful for predicting HLA subtypes in GD [73]. These investigations suggest matching different medical biotechnologies to better explain pathogenetic stages involving HLA haplotypes for the development of autoimmune disorders. 

By focusing on available molecular sources for CTSs, it appears that the parvo and polyoma viruses were investigated in the mCTSs. 

The role of viruses in inducing HT has been explored, but it is still not completely determined [52,74,75]. New data is becoming available regarding the roles of DNA and RNA viruses in triggering HT [76,77]. DNA viruses, namely, parvovirus 19 (B19V), human hepatitis C virus, and human herpes virus-6, have been associated with the viral pathogenesis of HT [76,77,78,79,80]. Among RNA viruses, human immunodeficiency virus (HIV) has been related to HT as it is able to activate the inflammatory immune response through IL-6 [81,82]. Particularly in HIV patients, this cytokine plays an important role by orchestrating the inflammatory cascade associated with HT [82]. The importance of IL-6 has been recognized even in animal models of DNA virus infection. In fact, IL-6 amounts are incremented in lung tissues of naïve Balb/c mice that received parvoviruses [83].

Parvoviruses are widespread in different countries on the American, European, and Asian continents [77]. Among DNA viruses, parvoviruses display the highest levels of replication and recombination [84]. These viruses can replicate autonomously or, conversely, recombine with a helper virus to be perpetuated [84]. The International Committee on Taxonomy of Viruses (ICTV) has reported members of the *Parvoviridae* family as small (~20 nm in diameter), icosahedral, non-enveloped viruses that have a small single-stranded DNA of 4–6 kb [85]. In 2020, the Executive Committee of the ICTV approved a revision for the taxonomy of the *Parvoviridae* family [86]. Although the definition to describe these viruses remained, genetic criteria used to demark members composing this family have been updated. The proposal criteria proceed from discoveries of new members of the *Parvoviridae* family through the application of HTS methods. Basically, the classification based on the association with the host has been abandoned because these viruses infect phylogenetically disparate hosts (see Table 1 in Ref. [86]) [86]. In this family, infectious agents for animals have been incorporated, showing a large host range. In fact, this is vast enough to include many phyla ranging from primates, mammals, and avian species to invertebrates [86]. Beyond this, the *Parvoviridae* family embraces pathogens for arthropod clades, namely the arachnids of the Chelicerata, that the molecular clock estimates go back to marine fossils of the late Cambrian period [87,88,89]. In 1975, Cossart and colleagues detected for the first-time B19V in serum samples of subjects screened for hepatitis B virus [90]. Thirty years later, Allander and colleagues discovered bocavirus 1 (HBoV1) in human samples of nasopharyngeal aspirates belonging to children with respiratory tract infections [91]. B19V1 may cause a widespread and self-limiting infection in children and adults, known as erythema infectiosum or fifth disease [92]. Both B19V and HBoV1 are pathogens for humans and have been detected in cancerous thyroid cells and HT lesions [76,93,94,95]. 

B19V and HBoV1 exhibit a particular tropism for the nuclear compartment. The host machinery for nuclear import of viral capsid is a critical step in the early phase of infection [96,97,98]. The capsid binding protein cleavage and polyadenylation specificity factor 6 plays a dominant role in directing integration to euchromatin of HBoV1 and lentivirus HIV-1, too [96,97,98]. During the later stages of infection, the replication of B19V leads to morphological changes in the nucleus. These are due to the spatial reorganization of chromatin that appears marginalized to the nuclear periphery by super-resolution microscopic examination [99]. 

In this investigation, MB have proved their worth in composing the future genetic makeup of individuals suffering from HT. This is because these methodologies were employed to disclose genetic susceptibility for HT in two molecular CTSs. Currently, several microsatellites have been proposed as significant elements to build up the molecular HT phenotypes. Specifically, heterozygous genotype Arg/Pro of rs 1042522 located on the TP 53 gene, polymorphisms of the IL-23R gene rs17375018, polymorphisms of the IL-6 gene promoter (-572) C/G, and IL-6 rs1800795 have been associated with HT susceptibility [100,101,102,103]. 

With the introduction of precision medicine in 2015, MB are considered instrumental in the management of cancerous lesions [104]. Molecular medicine has a key role in the diagnosis and treatment of thyroid cancers associated with HT by isolating molecular alterations in histological and cytological samples. In terms of histological fragments, the application of MB concerns the diagnosis of MALT lymphoma that develops around the primary HT alterations (see Table 1 in Ref. [105]) [105]. Genomic dissections of lymphomatous cells are employed to reveal the molecular phenotypes of MALT lymphoma. 

## 5. Conclusions

Decades of biomedical research on polymorphisms of HLA have revealed many genetic regions associated with HT. However, the epidemiological evidence related to HT diffusion cannot be fully explained by HLA genetic differences. This study sheds light on the requirement for a new linkage between MB and the production of data on demographic events such as births and migrations. This is because HH has not yet been proven and has been widely criticized but not clearly disproved.

Furthermore, in HT tissues, DNA viruses that cause mild manifestations of inflammatory diseases but produce nuclear DNA damage have been detected. Therefore, DNA viruses have relevance to HT pathogenesis and would offer important opportunities to develop antiviral strategies also able to treat HT. Mostly, viral infections should be considered in the future for the development and refinement of HT therapies for use as alternatives to or in conjunction with hormone replacement. 

Lastly, MB have enormous potential to promote precision medicine through the development of robust biomarkers to use for diagnosis and personalized therapies. 

## Figures and Tables

**Table 2:** Molecular clinical trial studies conducted on the HT population.

ClinicalTrials.gov Identifier	Target Sequences	Analysis and Methods	Biospecimen Genetic Retention and Description	Type and Model of Study	Time Perspective **	Enrollment of Subjects	Responsible Party	Results Overview
NCT03114267	Parvovirus	Analysis of the viral genome by PCR *, analysis of the presence of capsid protein	Not provided	Observational, cohort	Retrospective	64	Centre Hospitalier Universitaire, Amiens	No publications available
NCT03103776	Polyoma Virus	Positive PCR * frequencies for polyoma virus	Blood, Urine and/or Thyroid Tissue	Interventional, parallel assignment	NA	49	Centre Hospitalier Universitaire, Amiens	No publications available
NCT03447093	Oral microbiota	Measurement of microbiota by 16S rRNA gene.	Not provided	Observational, case-control	Cross-Sectional	120	First Affiliated Hospital of Harbin Medical University	Publications available
NCT03390582	Fecal microbiota	Measurement of microbiota by 16S rRNA gene.	Human feces	Observational, cohort	Cross-Sectional	200	First Affiliated Hospital of Harbin Medical University	No publications available
NCT00958113	HLA, CTLA4, thyroglobulin, THSR, CD40, PTPN2 and PTPN22	Map and identify genes that confer susceptibility to Autoimmune Thyroid Disease	Saliva	Observational, case-control	Cross-Sectional	199	University of Colorado, Denver, USA	No publications available
NCT02491567	CD40L, FOXP3, CTLA4, PTPN22, IL2RA, FCRL3 and HLADRB1	DNA methylation status of CpGs within gene promoters	Blood (leukocytes)	Observational, case-control	Cross-Sectional	110	Medical School of Aristotle University of Thessaloniki	Publications available

* PCR: polymerase chain reaction. ** NA: not available. Data taken from reference [46].

## Data Availability

Not applicable.
